# Supplementary material for: Multiple Timescale Dynamic Analysis of Functionally-Impairing Mutations in Human Ileal Bile Acid-Binding Protein
Source: Int J Mol Sci. 2022 Sep 26;23(19):11346. doi: 10.3390/ijms231911346 (PMC9569817; doi:10.3390/ijms231911346)
Supplement: Supplementary file 1 [file ijms-23-11346-s001.zip › ijms-1917371-supplementary.pdf]

Multiple Timescale Dynamic Analysis of Functionally-Impairing Mutations in Human Ileal Bile Acid-Binding Protein

Gergő Horváth [1], Bence Balterer [1], András Micsonai [2], József Kardos [2] and Orsolya Toke [1,*]

[1] NMR Research Laboratory, Centre for Structural Science, Research Centre for Natural Sciences, 2 Magyar tudósok körútja, H-1117 Budapest, Hungary
[2] ELTE NAP Neuroimmunology Research Group, Department of Biochemistry, Institute of Biology, ELTE Eötvös Loránd University, Pázmány Péter sétány 1/C, H-1117 Budapest, Hungary
* Correspondence: toke.orsolya@ttk.hu; Tel.: +36-1-382-6575

Supplementary Information

Table S1. Unfolding parameters of thermal denaturation experiments followed by CD

pH	WT		Q51A		Q99A	
	T_m (°C)	ΔH (kcal/mol)	T_m (°C)	ΔH (kcal/mol)	T_m (°C)	ΔH (kcal/mol)
5.8	58.4±0.1	85.4±2.0	57.2±0.1	83.7±1.4	57.5±0.1	87.3±1.9
6.3	58.9±0.1	80.9±1.7	57.3±0.1	79.5±1.9	57.9±0.1	82.1±1.6
8.0	59.0±0.1	89.4±2.0	56.8±0.1	90.5±2.1	57.5±0.1	82.7±1.8

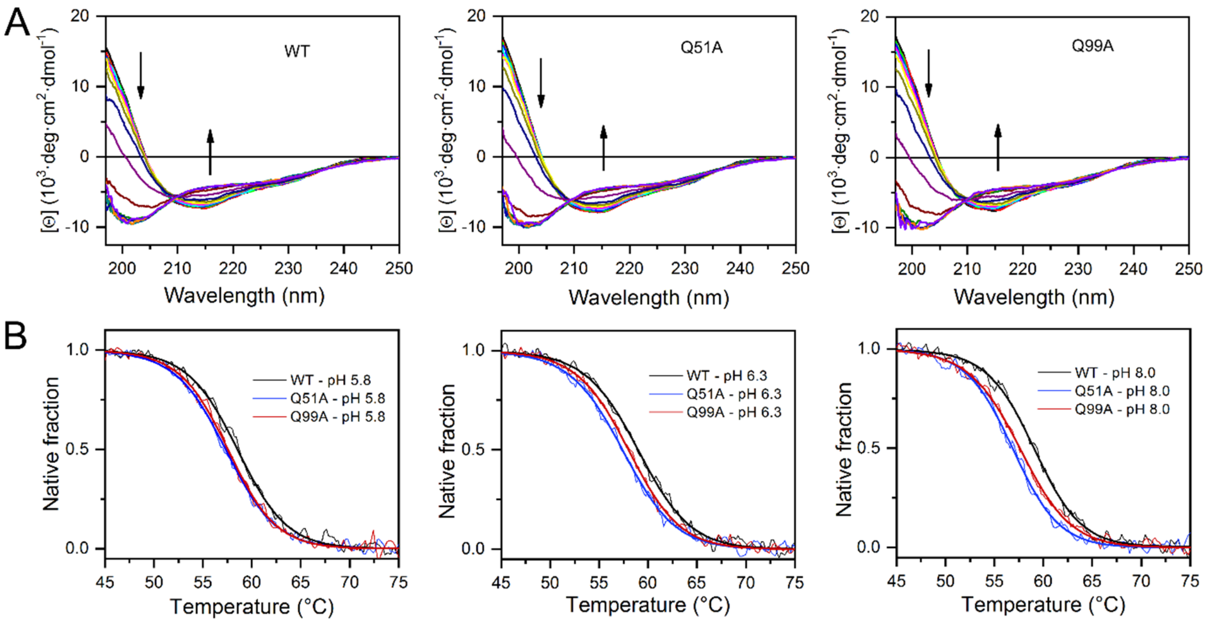

Figure S1. Thermal denaturation of *apo* human I-BABP variants followed by CD spectroscopy. (A) CD spectra were recorded at 5 °C steps from 20 to 85 °C. Arrows show the direction of changes. (B) Thermal denaturation of I-BABP variants using continuous heating with a rate of 2 °C/min was followed by the CD signal at 200 nm at pH 5.8, 6.3, and 8.0.

Table S2 Motional parameters derived from Model Free analysis of ^{15}N backbone relaxation measurements obtained for WT, Q51A, and Q99A human I-BABP in 20 mM K-phosphate, 50 mM KCl, 0.05% NaN_3, 10% D_2O, pH=6.3 at 283 K.

Residue	WT			Q51A			Q99A		
	S^2	R_{ex}	τ_e	S^2	R_{ex}	τ_e	S^2	R_{ex}	τ_e
**3**	0.91±0.02		1581±353	0.84±0.02		1691±291	0.86±0.02		1106±204
**4**	0.94±0.02			0.99±0.02			0.94±0.02		
**5**	0.84±0.02	1.8±0.3		0.92±0.02			0.88±0.02		
**6**	0.98±0.02			0.98±0.02			0.93±0.02		
**7**	0.97±0.02			0.92±0.02		1974±210	0.96±0.02		
**8**	0.98±0.02			0.98±0.02			N/A		
**9**	0.98±0.02			0.87±0.02		1855±307	0.93±0.02		
**10**	0.99±0.02			0.98±0.02			0.95±0.02		
**11**	0.95±0.02		1988±211	N/A			0.96±0.02		515±115
**12**	0.93±0.02			0.97±0.02			0.97±0.02		
**13**	0.84±0.02	1.1±0.2		0.80±0.02	1.7±0.3		0.85±0.02		
**14**	0.98±0.02			N/A			0.98±0.02		
**15**	0.91±0.02			0.94±0.02			0.95±0.02		
**16**	0.82±0.02	1.8±0.3		0.89±0.02			0.93±0.02		
**17**	0.86±0.02	2.1±0.3		0.94±0.02	2.1±0.3		0.96±0.02		
**18**	0.94±0.02			0.94±0.02			0.93±0.02		
**19**	0.89±0.02	1. 5±0.2		0.93±0.02			0.91±0.02		
**20**	0.80±0.02	3.4±0.3		0.86±0.02			0.90±0.02		
**21**	0.79±0.02	2.8±0.3		0.83±0.02	1.6±0.3		0.90±0.02		
**22**	0.82±0.02	1.3±0.2		0.82±0.02			0.85±0.02		
**23**	0.90±0.02			N/A			0.88±0.02		
**24**	0.89±0.02			0.87±0.02			0.89±0.02		
**25**	0.98±0.02			0.99±0.02			0.98±0.03		
**26**	0.91±0.02		1933±302	N/A			0.89±0.02		1839±239
**27**	0.89±0.02	1.5±0.3		0.89±0.02			0.90±0.02		
**28**	0.91±0.02	1.3±0.3		0.96±0.02			N/A		
**29**	0.86±0.02	3.4±0.3		N/A			0.95±0.03	3.1±0.3	
**30**	0.82±0.02	1.6±0.3		0.90±0.02	2.3±0.3		N/A		
**31**	0.90±0.02	2.8±0.3		0.99±0.02			N/A		
**32**	0.99±0.02	2.0±0.3		N/A			0.98±0.03	1.8±0.3	
**33**	0.98±0.02			0.85±0.02		1911±224	0.98±0.02		
**34**	0.98±0.02			0.89±0.02		1861±251	0.91±0.02		1104±181
**35**	0.89±0.02	4.3±0.3		0.98±0.02			0.86±0.03	5.9±0.4	
**36**	0.98±0.00	6.4±0.3		N/A			0.95±0.02		
**37**	0.98±0.02			0.94±0.02		1753±304	0.96±0.02		
**38**	0.97±0.02			0.88±0.02		1961±274	0.85±0.02		1904±361
**39**	0.98±0.02			0.97±0.02			0.91±0.02		1158±294
**40**	0.90±0.02	1.4±0.3		0.93±0.02			0.94±0.02		
**41**	0.91±0.02			0.99±0.02			N/A		
**42**	0.95±0.02			N/A			N/A		
**43**	0.91±0.02			0.93±0.02			N/A		
**44**	0.90±0.02			N/A			0.87±0.02		
**45**	0.83±0.02			0.85±0.02			0.83±0.02		
**46**	0.95±0.02			0.94±0.02			0.93±0.02		
**47**	0.94±0.02			0.97±0.02			0.93±0.02		
**48**	0.90±0.02			0.97±0.02			0.93±0.02		
**49**	0.98±0.02			0.98±0.02			1.00±0.02		
**50**	0.99±0.02			N/A			0.98±0.02		
**51**	0.95±0.02		1891±322	N/A			0.91±0.02		1961±255

**52**	0.99±0.02			0.98±0.02			0.98±0.03		
**53**	0.98±0.02			N/A			N/A		
**54**	0.85±0.02		1810±271	0.94±0.02			0.84±0.02		1443±191
**55**	0.98±0.02			0.98±0.02			0.99±0.03		
**56**	0.87±0.02		88±21	N/A			0.88±0.02		89±34
**58**	0.98±0.02			0.98±0.02			0.98±0.03		
**59**	0.87±0.02		1906±290	0.82±0.02		1881±244	0.85±0.02		1968±286
**60**	0.94±0.02		1518±243	0.82±0.04	1.1±0.2	1630±305	0.85±0.02		1755±251
**61**	0.97±0.02			0.89±0.02		1902±288	0.99±0.02		
**62**	0.79±0.02	1.4±0.3		0.88±0.02			0.90±0.02		
**63**	0.98±0.02			0.99±0.02			0.94±0.02		
**64**	0.98±0.02			0.87±0.02		1769±320	0.98±0.02		
**65**	0.93±0.02			0.94±0.02			0.95±0.02		
**66**	0.93±0.02			0.99±0.02			0.82±0.03	2.4±0.3	
**67**	0.96±0.02		1821±345	0.89±0.02		1922±341	0.98±0.02		
**68**	0.91±0.02			0.88±0.02		68±20	N/A		
**69**	0.88±0.02		1877±309	0.99±0.02			0.98±0.02		
**70**	N/A			N/A			0.64±0.03		2062±233
**71**	0.88±0.02			0.88±0.02			0.87±0.02		
**72**	0.85±0.02	1.4±0.2		0.92±0.02			0.95±0.02		
**73**	0.98±0.02			0.98±0.02			0.92±0.03	3.7±0.3	
**74**	0.99±0.02			0.98±0.02			N/A		
**75**	0.98±0.02			0.76±0.04	3.5±0.3	1918±256	0.98±0.02		
**76**	0.98±0.02			N/A			0.98±0.03		
**77**	0.75±0.03		544±108	0.67±0.03		828±93	0.74±0.03		596±95
**78**	0.92±0.02		124±32	N/A			0.90±0.02		123±21
**79**	0.86±0.02	1.2±0.3		0.96±0.02			0.90±0.02		
**80**	0.77±0.02	1.7±0.3					0.90±0.02		
**81**	0.89±0.02			0.87±0.02			0.93±0.02		
**82**	0.92±0.02			0.97±0.02			0.92±0.02		
**83**	0.89±0.02		1806±255	0.98±0.02			0.89±0.03		1913±208
**84**	0.83±0.02	1.3±0.2		0.89±0.02			0.92±0.02		
**85**	0.73±0.02	2.4±0.3		0.84±0.02			0.84±0.02		
**86**	0.83±0.02			0.86±0.02		59±21	0.85±0.02		
**87**	0.98±0.02			N/A			0.99±0.04		
**88**	0.96±0.02	2.1±0.3		0.98±0.02			0.96±0.03	3.9±0.3	
**89**	0.96±0.02			0.91±0.02		852±129	0.90±0.02		
**90**	0.93±0.02			0.98±0.02			0.99±0.02		
**91**	0.85±0.02	3.4±0.3		0.85±0.02	4.2±0.3		N/A		
**92**	0.96±0.02			0.99±0.02			0.86±0.03	1.8±0.3	
**93**	0.87±0.02			0.88±0.02			0.84±0.03	1.9±0.3	
**94**	0.90±0.02			0.96±0.02			0.84±0.02		
**96**	0.85±0.02	5.6±0.3		0.87±0.02	6.2±0.3		0.92±0.03	11.1±0.4	
**97**	0.88±0.02			0.90±0.02			0.79±0.03	4.4±0.3	
**98**	0.98±0.02	2.3±0.3		0.98±0.00	3.9±0.3		0.98±0.00	6.8±0.3	
**99**	0.98±0.02			0.98±0.02			0.98±0.02		
**100**	0.99±0.02			0.99±0.02			0.99±0.02		
**101**	0.89±0.02			0.98±0.02			0.97±0.02		
**102**	0.99±0.02			0.98±0.02			0.98±0.02		
**103**	0.92±0.02			0.87±0.02	1.3±0.2		0.91±0.02		
**104**	0.86±0.02	1.6±0.3		0.93±0.02			0.85±0.03	1.6±0.3	
**105**	0.98±0.02			0.98±0.02			0.98±0.02		
**106**	0.93±0.02			N/A			N/A		
**107**	0.91±0.02			0.92±0.02		121±29	0.89±0.02		
**108**	0.86±0.02	1.8±0.3		0.95±0.02		1349±208	0.92±0.02		
**109**	0.98±0.02			0.98±0.02			0.98±0.02		

**110**	0.99±0.02			0.98±0.02			0.98±0.03		
**111**	0.83±0.02			0.95±0.02			0.91±0.02		1613±
**112**	0.98±0.02			N/A			N/A		
**113**	0.98±0.02			0.98±0.02			0.98±0.02		
**114**	1.00±0.02			0.91±0.02		1907±259	N/A		
**115**	0.94±0.02			0.97±0.02			0.98±0.02		
**116**	0.98±0.02			0.98±0.02			0.98±0.02		
**117**	0.89±0.02			0.89±0.02			0.89±0.02		
**118**	0.93±0.02			0.91±0.02	1.1±0.2		0.98±0.02		
**119**	1.00±0.02			N/A			0.91±0.03	1.9±0.3	
**120**	0.99±0.02			0.96±0.02			0.88±0.03	2.6±0.3	
**121**	0.98±0.02			0.99±0.02			0.92±0.02		
**122**	0.98±0.02			0.98±0.02			0.98±0.02		
**123**	0.99±0.02			0.98±0.02			0.99±0.03		
**124**	0.97±0.02			0.98±0.02			0.96±0.02		
**125**	0.92±0.02			0.85±0.02		1133±256	0.92±0.02		
**126**	0.87±0.02			0.89±0.02			0.88±0.02		
**127**	0.68±0.03		1017±344	0.61±0.03		1423±418	0.63±0.03		1413±377

Table S3 Exchange parameters derived from global fit analysis of ^{15}N relaxation dispersion curves obtained for [80% ^{2}H, 99% ^{15}N-labeled] WT and Q99A human I-BABP in 20 mM K-phosphate, 50 mM KCl, 0.05% NaN_3, 10% D_2O, pH=6.3 at 283 K, 287 K, and 291 K. Parameters obtained for residues in cluster II in case of the wild-type protein are shown in blue.

**Residue**	**WT**<br>cluster I<br>T = 283 K k_{ex} = 836 ± 59 s^{-1}, p_b = 3.1 ± 0.2 %<br>T = 287 K k_{ex} = 1049 ± 88 s^{-1}, p_b = 3.9 ± 0.2 %<br>T = 291 K k_{ex} = 1540 ± 120 s^{-1}, p_b = 4.3 ± 0.2 %<br>cluster II<br>T = 283 K k_{ex} = 294 ± 40 s^{-1}, p_b = 1.8 ± 0.2 %<br>T = 287 K k_{ex} = 705 ± 69 s^{-1}, p_b = 2.0 ± 0.2 %<br>T = 291 K k_{ex} = 883 ± 103 s^{-1}, p_b = 2.2 ± 0.1 %					**Q99A**<br>T = 283 K k_{ex} = 871 ± 63 s^{-1}, p_b = 3.1 ± 0.2 %<br>T = 287 K k_{ex} = 1159 ± 57 s^{-1}, p_b = 3.5 ± 0.2 %<br>T = 291 K k_{ex} = 1546 ± 71 s^{-1}, p_b = 4.1 ± 0.2 %			
	***T* (K)**	***R*$_{ex}$ (Hz)**	***Φ* (Hz^2)**	***Δω* (Hz)**		***T* (K)**	***R*$_{ex}$ (Hz)**	***Φ* (Hz^2)**	***Δω* (Hz)**
E7	283	2.4±0.2	59±6	44±3		283			
	287	2.5±0.2	74±6			287			
	291	2.0±0.2	81±7			291			
E11	283	3.1±0.2	62±8	59±3		283			
	287	2.9±0.2	68±8			287			
	291	2.7±0.2	75±5			291			
N13	283	2.9±0.2	53±7	55±3		283			
	287	2.5±0.2	59±7			287			
	291	2.4±0.2	64±5			291			
F17	283	N/A	N/A	67±4		283			
	287	N/A	N/A			287			
	291	3.4±0.2	96±7			291			
K30	283	N/A	N/A	43±4		283			
	287	N/A	N/A			287			
	291	1.5±0.2	39±4			291			
K35	283	3.6±0.2	96±12	74±4		283			
	287	4.0±0.2	106±12			287			
	291	4.0±0.2	116±8			291			
I36	283	N/A	N/A	71±3		283			
	287	3.8±0.2	99±11			287			
	291	N/A	N/A			291			
T38	283	2.5±0.2	40±5	47±3		283			

	287	2.0±0.2	44±5			287			
	291	N/A	N/A			291			
E39	283	N/A	N/A	63±3		283			
	287	3.2±0.2	78±9			287			
	291	3.1±0.2	86±6			291			
V40	283	3.3±0.2	73±9	64±4		283			
	287	3.3±0.2	81±10			287			
	291	3.2±0.2	89±7			291			
H52	283	N/A	N/A	51±4		283			
	287	2.3±0.2	51±6			287			
	291	2.1±0.2	56±5			291			
Y53	283	N/A	N/A	52±4		283			
	287	2.4±0.2	54±7			287			
	291	N/A	N/A			291			
H57	283	3.5±0.2	85±11	69±4		283			
	287	3.7±0.2	94±11			287			
	291	3.6±0.2	103±8			291			
T58	283	3.6±0.3	89±11	68±3		283	1.1±0.2	41±4	37±3
	287	3.5±0.2	98±11			287	N/A	N/A	
	291	N/A	N/A			291	2.0±0.2	54±7	
M59	283	N/A	N/A	64±2		283			
	287	3.4±0.2	81±8			287			
	291	N/A	N/A			291			
T60	283	3.6±0.2	97±12	74±4		283	N/A	N/A	53±4
	287	4.1±0.2	108±12			287	N/A	N/A	
	291	4.0±0.2	118±8			291	2.6±0.3	110±10	
N61	283	3.4±0.2	82±10	68±3		283	2.5±0.3	64±8	46±5
	287	3.6±0.2	91±10			287	2.2±0.3	72±9	
	291	3.5±0.2	100±6			291	2.0±0.2	84±10	
K62	283	3.6±0.2	91±12	72±5		283	3.6±0.3	96±10	57±5
	287	3.9±0.3	101±12			287	3.6±0.2	109±11	
	291	3.8±0.2	111±9			291	3.3±0.3	126±13	
F63	283	2.3±0.2	36±5	45±3		283			
	287	1.8±0.2	40±5			287			
	291	1.7±0.2	44±4			291			
E68	283	N/A	N/A	75±5		283			
	287	N/A	N/A			287			
	291	5.4±0.3	234±19			291			
S69	283	N/A	N/A	44±3		283			
	287	2.5±0.2	74±6			287			
	291	N/A	N/A			291			
N70	283	N/A	N/A	55±3		283			
	287	N/A	N/A			287			
	291	2.9±	123±9			291			
I71	283	3.8±0.3	98±8	57±3		283	N/A	N/A	56±4
	287	4.0±0.3	122±9			287	N/A	N/A	
	291	3.2±0.2	134±9			291	2.9±0.2	122±11	
Q72	283	3.1±0.2	78±6	51±2		283	3.3±0.3	87±9	54±4
	287	3.3±0.2	98±6			287	3.0±0.3	99±9	
	291	2.6±0.2	107±7			291	2.7±0.2	115±10	
T73	283	14.4±0.2	677±49	150±5		283	8.3±0.3	271±27	95±7
	287	18.1±0.2	845±52			287	8.3±0.3	309±29	
	291	17.7±0.2	928±53			291	7.9±0.3	358±32	
M74	283	5.5±0.2	152±13	71±4		283			
	287	5.9±0.2	190±14			287			
	291	4.8±0.2	208±15			291			

	283	7.0±0.2	208±17		283	N/A	N/A	
T78	287	7.8±0.2	260±18	83±4	287	3.2±0.3	96±9	53±4
	291	6.5±0.2	285±19		291	2.6±0.2	111±10	
	283	5.0±0.2	137±11		283	N/A	N/A	
A81	287	5.4±0.3	171±12	68±3	287	3.1±0.2	91±10	52±4
	291	4.4±0.2	187±12		291	3.0±0.3	106±11	
	283	3.4±0.2	86±6		283	3.7±0.3	98±11	
T82	287	3.6±0.2	107±7	54±2	287	3.4±0.3	112±12	57±5
	291	2.8±0.2	118±7		291	3.1±0.2	129±13	
	283				283	N/A	N/A	
V83	287				287	N/A	N/A	62±4
	291				291	3.6±0.3	152±12	
	283	5.5±0.2	155±12		283	4.8±0.3	132±13	
G88	287	6.0±0.3	193±13	72±3	287	4.4±0.3	151±14	67±5
	291	4.9±0.2	212±13		291	4.1±0.3	175±16	
	283				283	N/A	N/A	
K89	287				287	N/A	N/A	54±4
	291				291	3.3±0.3	116±10	
	283	2.5±0.2	61±6		283			
L90	287	2.6±0.2	76±6	45±3	287			
	291	N/A	N/A		291			
	283	16.6±0.2	953±65		283	N/A	N/A	
V91	287	21.8±0.2	1189±67	178±4	287	N/A	N/A	60±4
	291	22.6±0.2	1305±67		291	3.4±0.3	144±12	
	283	N/A	N/A		283	4.6±0.3	129±13	
V92	287	2.7±0.3	80±7	46±3	287	4.3±0.2	147±14	66±5
	291	N/A	N/A		291	4.0±0.3	170±15	
	283	N/A	N/A		283			
N93	287	3.9±0.2	117±10	56±4	287			
	291	3.1±0.2	129±11		291			
	283	N/A	N/A		283			
F94	287	N/A	N/A	48±3	287			
	291	2.3±0.2	95±7		291			
	283	11.4±0.2	431±35		283			
N96	287	13.6±0.3	537±38	120±6	287			
	291	12.3±0.2	590±40		291			
	283	6.6±0.2	193±16		283	5.3±0.3	152±13	
Y97	287	7.3±0.2	241±17	80±4	287	5.0±0.3	174±14	71±4
	291	6.1±0.2	265±18		291	4.6±0.2	201±15	
	283	10.5±0.2	376±30		283	N/A	N/A	
H98	287	12.3±0.2	469±32	112±5	287	4.4±0.3	151±12	67±4
	291	11.0±0.2	515±33		291	4.1±0.2	174±13	
	283	6.2±0.2	179±15		283			
Q99/A99	287	6.8±0.2	223±16	77±4	287			
	291	5.6±0.2	245±17		291			
	283	5.3±0.2	147±11		283	9.6±0.3	331±29	
T100	287	5.8±0.2	183±12	70±3	287	9.7±0.3	377±30	105±6
	291	4.7±0.3	201±13		291	9.4±0.3	436±33	
	283	4.8±0.2	130±10		283	2.5±0.3	64±7	
S101	287	5.2±0.2	162±11	66±3	287	2.3±0.3	73±8	46±4
	291	4.2±0.2	177±12		291	2.0±0.2	85±8	
	283	3.3±0.2	84±7		283	2.4±0.3	61±6	
E102	287	3.5±0.2	105±8	53±3	287	2.2±0.2	70±6	45±3
	291	2.7±0.2	115±8		291	1.9±0.2	81±7	
G105	283	N/A	N/A	52±2	283			
	287	N/A	N/A		287			

	291	2.6±0.2	110±7			291			
	283	3.5±0.2	90±9			283			
V109	287	3.7±0.2	112±10	55±4		287			
	291	2.9±0.2	123±11			291			
	283	3.7±0.2	96±8			283			
E110	287	3.9±0.2	120±9	57±3		287			
	291	3.1±0.2	132±9			291			
	283					283	N/A	N/A	
V111	287					287	N/A	N/A	66±4
	291					291	4.0±0.3	172±13	
	283	2.2±0.2	54±5			283	2.0±0.2	51±5	
T113	287	2.3±0.2	68±6	43±3		287	1.8±0.2	58±5	41±3
	291	1.8±0.2	75±6			291	1.6±0.2	67±6	
	283	2.6±0.2	64±6			283			
G115	287	2.7±0.3	80±7	46±3		287			
	291	2.1±0.2	88±7			291			
	283	3.1±0.3	78±8			283	N/A	N/A	
Y119	287	3.3±0.2	98±9	51±4		287	3.6±0.2	113±10	57±4
	291	2.6±0.2	107±10			291	4.0±0.4	128±14	
	283	3.8±0.3	98±8			283	3.7±0.3	98±9	
R121	287	4.0±0.3	122±9	57±3		287	N/A	N/A	57±4
	291	N/A	N/A			291	N/A	N/A	
	283	N/A	N/A			283	N/A	N/A	
V122	287	N/A	N/A	55±2		287	3.4±0.2	113±10	58±4
	291	3.0±0.2	126±7			291	N/A	N/A	

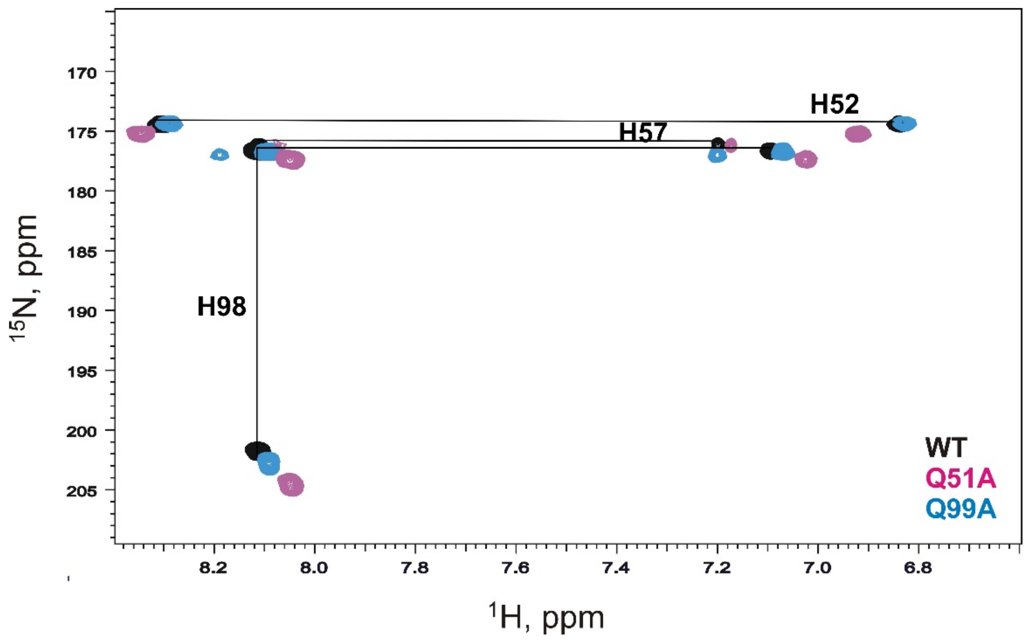

Figure S2. ^{1}H-^{15}N long-range HSQC spectra of WT, Q51A, and Q99A *apo* human I-BABP at pH 6.3, 10 °C correlating histidine $^{15}N_{\delta1}$ and $^{15}N_{\varepsilon2}$ chemical shifts with nonexchangeable C-H ring protons. The similar pattern of chemical shifts obtained for the three variants indicates no significant mutation-induced change in the tautomer equilibria of histidines.
